# Specificity in PDZ-peptide interaction networks: Computational analysis and review

**DOI:** 10.1016/j.yjsbx.2020.100022

**Published:** 2020-03-07

**Authors:** Jeanine F. Amacher, Lionel Brooks, Thomas H. Hampton, Dean R. Madden

**Affiliations:** a Department of Biochemistry and Cell Biology, Geisel School of Medicine at Dartmouth, Hanover, NH 03755, USA; b Department of Chemistry, Western Washington University, Bellingham, WA 98225, USA; c Department of Biology, Western Washington University, Bellingham, WA 98225, USA; d Department of Microbiology and Immunology, Geisel School of Medicine at Dartmouth, Hanover, NH 03755, USA

**Keywords:** Protein-protein interactions, PDZ, Peptide-binding domains, Therapeutic targets

## Abstract

Globular PDZ domains typically serve as protein–protein interaction modules that regulate a wide variety of cellular functions via recognition of short linear motifs (SLiMs). Often, PDZ mediated-interactions are essential components of macromolecular complexes, and disruption affects the entire scaffold. Due to their roles as linchpins in trafficking and signaling pathways, PDZ domains are attractive targets: both for controlling viral pathogens, which bind PDZ domains and hijack cellular machinery, as well as for developing therapies to combat human disease. However, successful therapeutic interventions that avoid off-target effects are a challenge, because each PDZ domain interacts with a number of cellular targets, and specific binding preferences can be difficult to decipher. Over twenty-five years of research has produced a wealth of data on the stereochemical preferences of individual PDZ proteins and their binding partners. Currently the field lacks a central repository for this information. Here, we provide this important resource and provide a manually curated, comprehensive list of the 271 human PDZ domains. We use individual domain, as well as recent genomic and proteomic, data in order to gain a holistic view of PDZ domains and interaction networks, arguing this knowledge is critical to optimize targeting selectivity and to benefit human health.

## Highlights

• A list of the PDZ domains in the human proteome, including associated names and other domains.
• A list of all mammalian PDZ domain-containing entries (total: 468) in the PDB.
• Statistical analyses of binding affinities from for both endogenous and engineered sequences.
• An extensive review of the PDZ selectivity literature over the past 25 years.

## Graphical abstract

**Figure:**
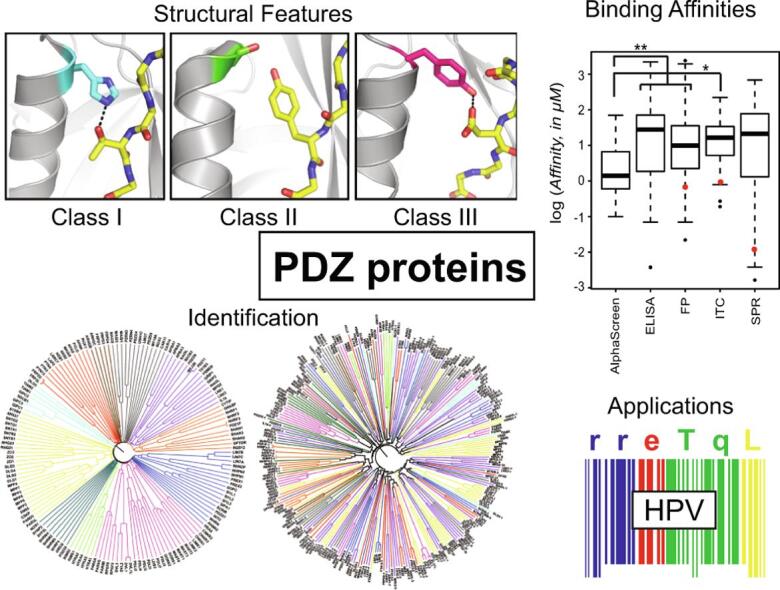

## 1 Introduction

Stereochemical complementarity is the foundation of molecular recognition. It regulates the formation of protein–protein interactions that govern post-translational modification, trafficking, and localization. In addition to controlling overall molecular activity, the resulting changes in protein chemistry, concentration, and assembly can reciprocally modulate the free-energy landscape of each interaction in its specific physiological context, creating both feed-back and feed-forward systems. Furthermore, the probability of formation of an individual protein–protein interaction *in vivo* is co-determined by the other potential binding partners in the cell. Since mutagenesis often affects more than one of these competing factors, it can be difficult to deconvolute the role of pair-wise specificities in controlling the biological read-out of a particular interaction.

This issue is particularly acute in the case of peptide-recognition domains (PRDs), which form the basis for many trafficking and signaling networks within the cell. These domains recognize cognate short linear motifs (SLiMs) – conserved peptide sequence patterns that reflect the stereochemical requirements of corresponding binding pockets in the PRD. Through evolutionary diversification, PRDs typically occur in large families whose distinct binding preferences are driven by sequence variations along the length of the peptide-binding site. However, multiple PRDs may have affinity for a shared target due to incomplete diversification and/or for importance in regulation of biological processes. Conversely, a single PRD often recognizes multiple targets. These domains are often found in tandem with other PRD or functional components, creating a combinatorial mosaic of possible interactions for such multidomain scaffolding proteins. Thus, understanding or manipulating a particular PRD-target interaction often requires detailed biochemical characterization not only of the primary interaction, but also of others within a shared network.

The most abundant PRD family in the human genome was first identified based on a shared “GLGF” sequence motif (Ponting, 1997, Songyang, 1999). It is now referred to as the PDZ family, named for the earliest recognized examples: PSD-95, a 95kD protein from the post-synaptic density (also called DLG4); Dlg, the *Drosophila* discs-large-1 tumor suppressor protein; and ZO-1, the epithelial tight-junction protein zonula occludens 1 (Bryant et al., 1993, Cho et al., 1992, Kennedy, 1995, Woods and Bryant, 1991, Woods and Bryant, 1989). PDZ sequences are found across the phylogenetic tree in mammals, yeast, plants, and bacteria (Ponting, 1997). Through multivalent scaffolding, PDZ proteins can drive the formation of functional microdomains, particularly for targets that include membrane-bound receptors and ion channels (Harris and Lim, 2001, Nourry et al., 2003). They can also serve as adaptors, connecting receptors to cytoskeletal elements that regulate cellular trafficking (Bunn et al., 1999, Ye and Zhang, 2013). Underscoring their functional importance, several PDZ proteins are strictly required for normal organismal development (e.g., Bladt et al., 2002, Xu et al., 2008). Others are actively sabotaged by viral pathogens. For example, the E6 proteins of oncogenic human papillomavirus (HPV) strains contain binding motifs that can act as competitive agonists for diverse sets of PDZ domains (Lee and Laimins, 2004, Nguyen et al., 2003, Pim et al., 2012).

PDZ domains typically bind peptides located at the extreme C-terminus of target proteins, engaging the terminal carboxylate moiety with backbone amide groups positioned within a loop formed by GLGF sequence homologs. The C-terminal (or P^0^) side chain is bound in an adjacent pocket, whose binding preference is determined not only by the PDZ side chains that line it, but also by the spacing relative to the loop (Amacher et al., 2013, Harris and Lim, 2001, Songyang et al., 1997). Many PDZ domains also engage in conserved interactions with the P^−2^ side chain located two amino acids closer to the N-terminus. Indeed, the identity of the C-terminal and P^−2^ residues formed the basis for the earliest classification of PDZ domains into three groups (Songyang et al., 1997).

In addition to their binding promiscuity, individual PDZ domain interactions are often highly dynamic, and kinetic experiments reveal relatively high off-rates and correspondingly weak affinities (Gianni et al., 2005, Haq et al., 2012, Ivarsson, 2012). This may be because the affinity and kinetics of the individual interactions have been tuned to facilitate the cargo 'hand-offs' required for efficient trafficking. Likewise, it may be because a given PDZ:peptide interaction is only one component in a larger complex, whose stability is regulated by local concentration and multidentate scaffolding associations. In either case, even very weak PDZ:peptide affinities (*K*_D_ > 100 μM) can underpin physiologically significant interactions (e.g., Cushing et al., 2008).

Due to their central role in scaffolding macromolecular complexes critical to signaling and trafficking pathways, PDZ domains are active therapeutic targets in multiple human diseases, including: drug and alcohol addiction (MPDZ), stroke, pain, epilepsy, and depression (PSD-95, PATJ), cystic fibrosis (CAL, NHERF2, GRASP), neurodegenerative disorders (PICK1), and various cancers (Dvl, Tiam1, GIPC) (Bach et al., 2012, Cook et al., 2012, Cui et al., 2007, Cushing et al., 2010, Gee et al., 2011, Grandy et al., 2009, Houslay, 2009, Kundu et al., 2012, Liu et al., 2013, Patra et al., 2012, Shepherd et al., 2010, Tao and Johns, 2006, Thorsen et al., 2010, Vouilleme et al., 2010, Zhang et al., 2011a, Zhang et al., 2011b, Zhang et al., 2009). Identifying a therapeutic window for complex-specific drug design is a multilayered problem, however, requiring a detailed understanding of a number of interrelated protein–protein interactions: those of the PDZ:target itself, as well as other PDZ domains with the same target and the PDZ domain with its additional interacting partners.

A large majority of the available information regarding PDZ domain function has focused on individual domain data. Specifically, work on founding members PSD-95 and Dlg1, as well as Erbin and NHERF1, among others, identified key characteristics, such as known targets, posttranslational modifications, structure, and binding affinities (e.g., Cushing et al., 2008, Doyle et al., 1996, Skelton et al., 2003, Tonikian et al., 2008). While these studies have elucidated many general principles of PDZ-mediated interactions, the field currently lacks a central resource for this data. In addition, a number of informative reviews address the cellular roles, known binding targets, and general characteristics of PDZ domains (specifically, Harris and Lim, 2001, Lee and Zheng, 2010, Nourry et al., 2003), but the last decade of large-scale experiments and available genomic and proteomic database information provide new perspectives on how PDZ domains engage their targets. In this review, we bring this information together for the first time, arguing that knowledge of these biochemical parameters is necessary to understand multivalent interactions involving PDZ domains, and may aid in efficacious therapy development.

### 1.1 UniProt, Pfam, and SMART, oh my! defining the scope of the PDZome

Estimates of the number of PDZ domains in the human proteome have ranged from 250 to over 400, depending on the database and method of identification used, with a general consensus of approximately 270 (e.g., refs. Houslay, 2009, Hui et al., 2013, Luck et al., 2012, te Velthuis et al., 2011). Using hidden Markov models to identify sequence profiles, the Pfam database reports 247 human PDZ domains (Table S1) (El-Gebali et al., 2019). Conversely, the SMART (Simple Modular Architecture Research Tool) database reports 666 human proteins containing 1101 PDZ domains (Schultz et al., 1998). However, the SMART database includes alternatively spliced protein isoforms as distinct entries, leading to considerable redundancy. UniProt (the Universal Protein Resource) avoids redundancy by clustering isoforms, and 'crowdsources' domain identification by combining information from Pfam, SMART and the PROSITE database, identifying a total of 274 human PDZ domains in 155 proteins (Table S1) (UniProt Consortium, 2019, UniProt Consortium, 2012).

Since automated sequence-based domain identification often involves trade-offs between stringency and completeness, the development of a more definitive PDZ database requires manual curation. In order to determine the exact number of human PDZ domains, we used our knowledge of the conserved structure of this domain. The first structure to be determined was that of the third PDZ domain of PSD-95, using X-ray crystallography (Doyle et al., 1996). Doyle *et al*. used the rat sequence in their study; however, the only difference between the human and rat sequences is a single amino-acid substitution, V328I. This structure and many others highlight the characteristic fold of PDZ domains, which are typically 80–100 residues in length, and contain a core of 5 β-strands (βA-βE) and 2α–helices (αA and αB) (Lee and Zheng, 2010, Ponting, 1997). The exact number of secondary structure elements in PDZ domains can be higher (e.g., TIP-1 [PDB entry code: 4SFJ], USH1C-1 [3KIR], and MPDZ-13 [2FNE]).

We therefore superimposed an alignment of the location of the canonical PDZ secondary structure elements onto a sequence alignment of all proposed domains. We also took into consideration whether or not a putative domain contains the conserved carboxylate-binding loop sequence. Of the domains listed by UniProt, 274 contain all required secondary structure elements. However, the carboxylate-binding loop sequence is missing in first PDZ domain of the FERM and PDZ domain-containing protein 2B pseudogene, FRP2L, suggesting that it is not a canonical PDZ domain. In addition, CNIPF is a fusion protein of CNKR3 and IPCEF1, and its PDZ domain sequence is 100% identical with that of CNKR3. Therefore, in analogy to the splice variants mentioned above, we do not consider this to be a unique occurrence of this PDZ domain. Finally, structural alignments identified two candidate PDZ domains that are flagged as distinct in the UniProt list, each representing the second PDZ domain in one of the two Golgi reassembly-stacking proteins GORS1 and GORS2. Although the overall fold is intact, structural data previously revealed a very unusual secondary structure layout, in that the βA and βB strands are at the C-terminal end of the domain, connected to the βE strand (Truschel et al., 2012, Truschel et al., 2011). These PDZ domains show the highest structural similarity to prokaryotic PDZ domains, and reflect an example of circular permutation, whereby the C-terminus of a protein is shifted, so that secondary structural elements are out-of-order (Hultqvist et al., 2013, Hultqvist et al., 2012, Truschel et al., 2011). Thus, our manually curated database includes a final total of 154 human proteins, containing 272 unique PDZ domains. The complete list of protein is reported in Table S1, along with alternative nomenclatures. PDZ domains are listed in Table 1, and corresponding sequences can be found in UniProt.

**Table 1:** Structural Coverage of PDZ domains in the Protein Data Bank (PDB). Individual PDB entries associated with each domain are reported in Table S2. Green text: a structure has been determined for a human domain or for a rodent domain with >70% sequence homology to the corresponding human domain.

**AFAD**	**EM55**	IL16-2	**MAGI2-4**	**NHRF2-2**	PDZD8	**SHAN1**

**AHNK2**	**ERBIN**	**IL16-3**	**MAGI2-5**	**NHRF3-1**	**PDZD9**	SHAN2
AHNK	FRP2L-2	**IL16-4**	**MAGI2-6**	**NHRF3-2**	**PICK1**	**SHAN3**
**APBA1-1**	**FRPD1**	**INADL-1**	MAGI3-1	**NHRF3-3**	**PRAX**	SHRM2
**APBA1-2**	FRPD2-1	**INADL-2**	MAGI3-2	**NHRF3-4**	**PREX1**	SHRM3
**APBA2-1**	**FRPD2-2**	**INADL-3**	**MAGI3-3**	NHRF4-1	PREX2-1	**SHRM4**
**APBA2-2**	FRPD2-3	INADL-4	MAGI3-4	NHRF4-2	PREX2-2	SI1L1
**APBA3-1**	FRPD3	**INADL-5**	MAGI3-5	**NHRF4-3**	PSMD9	SI1L2
**APBA3-2**	FRPD4	**INADL-6**	MAGI3-6	NHRF4-4	**PTN13-1**	SI1L3
**ARHGB**	**GIPC1**	**INADL-7**	**MAGIX**	**NOS1**	**PTN13-2**	SIPA1
**ARHGC**	**GIPC2**	**INADL-8**	**MAST1**	PAR3L-1	**PTN13-3**	**SNTA1**
CAR11	GIPC3	**INADL-9**	**MAST2**	PAR3L-2	PTN13-4	SNTB1
CAR14	**GOPC**	INADL-10	**MAST3**	**PAR3L-3**	PTN13-5	**SNTB2**
CNKR1	**GORS1-1**	INTU	**MAST4**	PAR6A	**PTN3**	SNTG1
CNKR2	**GORS1-2**	**LDB3**	**MPDZ-1**	**PAR6B**	**PTN4**	SNTG2
CNKR3	**GORS2-1**	LIMK1	MPDZ-2	PAR6G	**PZRN3-1**	**SNX27**
**CSKP**	**GORS2-2**	**LIMK2**	**MPDZ-3**	PARD3-1	**PZRN3-2**	**STXB4**
**CYTIP**	**GRASP**	LIN7A	**MPDZ-4**	**PARD3-2**	PZRN4-1	**SYJ2B**
**DLG1-1**	GRD2I-1	**LIN7B**	MPDZ-5	**PARD3-3**	PZRN4-2	SYNP2
**DLG1-2**	GRD2I-2	LIN7C	MPDZ-6	**PCLO**	**RADIL**	SYP2L
**DLG1-3**	**GRIP1-1**	**LMO7**	**MPDZ-7**	**PDLI1**	**RGS12**	**TIAM1**
**DLG2-1**	**GRIP1-2**	LNX1-1	**MPDZ-8**	**PDLI2**	**RGS3**	TIAM2
**DLG2-2**	GRIP1-3	**LNX1-2**	MPDZ-9	**PDLI3**	**RHG21**	**TX1B3**
**DLG2-3**	**GRIP1-4**	**LNX1-3**	**MPDZ-10**	**PDLI4**	RHG23	**USH1C-1**
**DLG3-1**	**GRIP1-5**	LNX1-4	**MPDZ-11**	**PDLI5**	RHN2L	**USH1C-2**
**DLG3-2**	**GRIP1-6**	LNX2-1	**MPDZ-12**	**PDLI7**	RHPN1	**USH1C-3**
**DLG3-3**	**GRIP1-7**	**LNX2-2**	**MPDZ-13**	**PDZ11**	**RHPN2**	**WHRN-1**
**DLG4-1**	GRIP2-1	LNX2-3	**MPP2**	PDZ1P-1	**RIMS1**	**WHRN-2**
**DLG4-2**	GRIP2-2	LNX2-4	MPP3	PDZ1P-2	**RIMS2**	**WHRN-3**
**DLG4-3**	**GRIP2-3**	LRRC7	MPP4	PDZ1P-3	RPGF2	**ZO1-1**
DLG5-1	**GRIP2-4**	MAGI1-1	**MPP5**	PDZD2-1	RPGF6	**ZO1-2**
DLG5-2	GRIP2-5	**MAGI1-2**	MPP6	PDZD2-2	**SCRIB-1**	**ZO1-3**
**DLG5-3**	GRIP2-6	**MAGI1-3**	**MPP7**	PDZD2-3	**SCRIB-2**	**ZO2-1**
DLG5-4	GRIP2-7	**MAGI1-4**	MY18A	PDZD2-4	**SCRIB-3**	**ZO2-2**
DPTOR	**HTRA1**	MAGI1-5	**NEB1**	PDZD2-5	**SCRIB-4**	ZO2-3
**DVL1**	**HTRA2**	MAGI1-6	**NEB2**	PDZD2-6	**SDCB1-1**	ZO3-1
**DVL2**	**HTRA3**	MAGI2-1	**NHRF1-1**	PDZD4	**SDCB1-2**	ZO3-2
DVL3	HTRA4	**MAGI2-2**	**NHRF1-2**	**PDZD7-1**	SDCB2-1	ZO3-3
DVLP1	IL16-1	**MAGI2-3**	**NHRF2-1**	PDZD7-2	SDCB2-2	

**Fig. 1:**
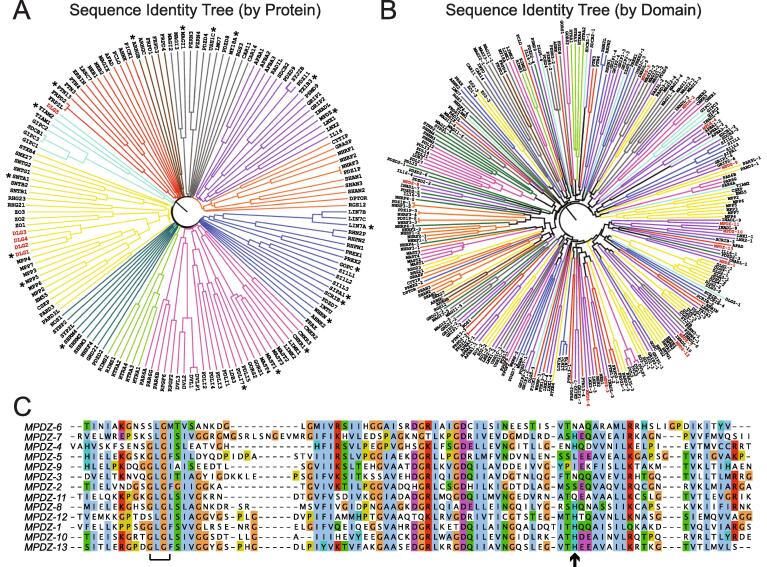
Sequence similarity in PDZ domain-containing proteins. (A–B) Sequence identity trees of human PDZ domain-containing proteins (A) or PDZ domains (B), where the hyphenated number distinguishes among multiple PDZ domains within a single protein (e.g., NHERF1-1 and NHERF1-2 are the first and second PDZ domains in the NHERF1 protein). Sequences were downloaded from UniProt, identity was assessed using CLUSTALW, and trees were generated using PHYLIP. Branch colors in both (A) and (B) are based on the nearest neighbors of a single node in (A). Asterisks in (A) highlight proteins displayed in Fig. 2, Fig. 3. Red labels highlight DLG family proteins (1–5) in (A) and MPDZ PDZ domains (1–13) in (B). (C) Alignment of the 13 PDZ domains of MPDZ. Sequence elements that determine peptide specificity are indicated beneath the sequences, specifically the carboxylate-binding loop (bracket) and the αB-1 residue (arrow). (For interpretation of the references to colour in this figure legend, the reader is referred to the web version of this article.)

To investigate sequence relationships within this group of proteins, we used CLUSTALW and PHYLIP (Felsenstein, 1988, Larkin et al., 2007) to cluster the human PDZ domain-containing proteins by sequence identity, with the exception of GORS1-2 and GORS2-2, since the PDZ sequences are so distinct. The resulting tree, colored by central nodes (Fig. 1A), recapitulates well-established homologies, e.g., among the ZO or LIN7 family members. However, some family members show more distant relationships, such as Dlg5, which is clearly separated from the cluster containing Dlg1–4 (highlighted in red in Fig. 1A). Of course, whole-protein sequence alignments reflect not only identity among individual PDZ domains, but also the arrangement of multiple domains that are often found in tandem.

**Fig. 2:**
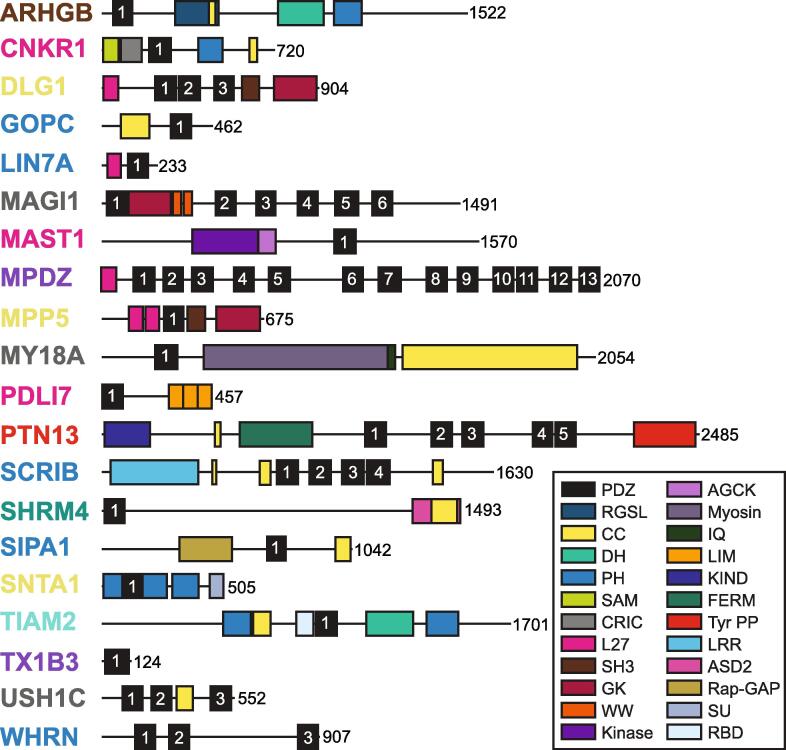
Variable domain architecture of 20 PDZ domain-containing proteins. All proteins are labeled according to UniProt designations, with the color of the name matching the color of the corresponding node in Fig. 1A. The proteins are drawn to scale according to the the number of residues (shown to the right of each sequence). PDZ domains are shown as black rectangles and are numbered within each protein. Other domains are color coded as described in the key. (For interpretation of the references to colour in this figure legend, the reader is referred to the web version of this article.)

To get a better idea of the sequence relationships between individual PDZ domains, we aligned just the PDZ domain sequences and used CLUSTALW and PHYLIP to create a tree (Fig. 1B). In this PDZ domain tree, we chose to retain the protein branch color-coding from our whole-protein sequence tree (Fig. 1A). Here, we see that PDZ domains from a given protein family often do not cluster together. To highlight these differences, we aligned the 13 PDZ domains of MPDZ (Fig. 1C). There are two critical sequence features that suggest differences in target specificity amongst these domains, corresponding to their dispersion across the PDZome (Fig. 1B, names in red). First, the final position of the carboxylate-binding loop sequences (the “GLGF” motif) in the domains contain a variety of amino acids, including Met, Ile, Phe, and Leu. The identity of this amino acid determines whether or not a PDZ domain can accommodate a P^0^ Ile, as discussed in the next section (Amacher et al., 2013). Second, the first position of the αB helix, termed αB-1, determines P^−2^ selectivity, and the binding class of the PDZ domain. Again, in the 13 MPDZ PDZ domains we see multiple amino acids at this residue: while 7 of the domains contain a Class I-determining His, others have an Asn, Glu, Ile, or Leu at this position (Fig. 2C), characteristic of different class selectivity (Songyang et al., 1997, Stiffler et al., 2007).

We were also curious how the number of PDZ domains varies in the 154 PDZ domain-containing proteins. Considering just the PDZ domains, 111 (or 72%) of the proteins contain 1 PDZ domain, while 43 (or 28%) contain 2 or more PDZ domains (Table S1). The number of PDZ domains range from 1 to 13 (in MPDZ). Often, there are other modular protein domains also present in these proteins, e.g., SH3 or guanylate kinase, which can be similarly identified by consensus sequence alignments (Marchler-Bauer et al., 2013).

**Fig. 3:**
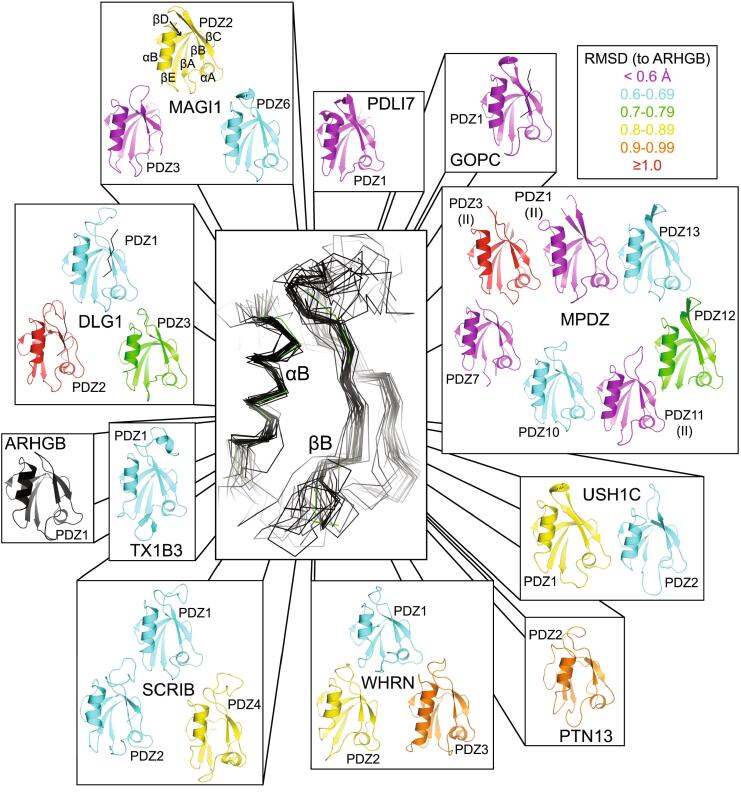
Conservation of the PDZ domain fold. Of the 20 PDZ domain-containing proteins in Fig. 2, 11 have associated structures, which include a total of 26 PDZ domains. We aligned the βB strand and αB helix of each to our reference structure, ARHGB PDZ (PDB ID: 2DLS), with RMSD values ranging from 0.39 Å (MPDZ-1; 2O2T) to 1.37 (MPDZ-3; 2IWN), and an average of 0.72 Å. The central box shows an alignment of all 26 peptide-binding clefts, represented by C_α_ traces. The individual PDZ domain structures are clustered by parent protein in the boxes surrounding the center alignment, and are colored based on RMSD to the peptide-binding cleft of ARHGB PDZ (see key). Most of the PDZ domains are classified as canonical Class I PDZ domains, with the exceptions of MPDZ PDZ1, 3, and 11, as labeled in brackets underneath the domain name. Binding classification does not seem to determine overall fold, as both MPDZ PDZ1 and 11 show minimal differences to ARHGB PDZ (RMSD < 0.6 Å). The conserved secondary structure elements are labeled in the top left box, on the MAGI1-2 structure. PDB IDs are: 1PDR, 1UEZ, 1UF1, 1UFX, 1UJU, 1WHA, 1X5N, 2DLS, 2FCF, 2FNE, 2IWN, 2IWP, 2KPK, 2O2T, 2OPG, 2OQS, 2Q3G, 2QG1, 2R4H, 2W4F, 3BPU, 3DJI, 3KIR, 3PDZ, 3RL7, 4E34.

We selected 20 PDZ domain-containing proteins (asterisks in Fig. 1A), in order to highlight the variety in domain architecture, and to complement structures illustrated in other reviews (Kim and Sheng, 2004, Lee and Zheng, 2010, Manjunath et al., 2018, Nourry et al., 2003, Ye and Zhang, 2013). The domain layouts for these proteins are shown schematically in Fig. 2. The individual proteins contain as few as one and as many as 13 PDZ domains. Structures are available for just over half of the collective total of 50 PDZ domains. The central panel in Fig. 3 shows C_a_ traces for these 26 available PDZ structures following least-squares superposition of the βB strand and αB helix onto the Rho guanine nucleotide exchange factor 11 (ARHGB) domain. The root-mean-square deviation (RMSD) values are all less than <1.4 Å, independent of binding-motif designation. Areas of tight clustering correspond to the conserved architecture of the domain core around the peptide-binding cleft, in contrast to regions of high structural variability encoded in loop regions. In the surrounding panels, ribbon diagrams for the individual domains are clustered by protein of origin and colored by RMSD of the superposition, permitting visualization of individual adaptations.

### 1.2 Variations on a theme: the conserved structure of the PDZ domain

Many PDZ domains readily crystallize and hundreds of PDZ domain structures are publicly available. To identify them as comprehensively as possible, we developed a Python-based sequence-matching algorithm utilizing BLASTP (Altschul et al., 1990). Using 70% sequence identity as a cut-off for all identifications, the Protein Data Bank (PDB) contains 471 entries that include one or more PDZ domain structures (Table 1; Table S2) (Bernstein et al., 1977). This cut-off includes structures of PDZ domains derived from mouse or rat proteins (in red in Table S2). Manual validation checks confirmed that using this sequence-identity cut-off, we also identify a small number of *Xenopus tropicalis* (western clawed frog) and *Drosophila melanogaster* (fruit fly) PDZ structures, but these sequences have not been included in our table. Searches with a <70% identity cut-off also identify bacterial PDZ domains.

The set of 471 entries include a total 505 unique structures of PDZ domains. Based on this set, at least one experimental structure has been determined for 163 of the 271 human PDZ domains. The structurally characterized domains belong to 102 of the 154 distinct human proteins containing at least one PDZ domain. Some domains have been extensively characterized: there are 30 distinct PDB entries including structures of DLG4-3, and 29 of the GOPC domain (Table S2). We note that this propensity of many PDZ domains to crystallize makes them well-suited for technical studies. For example, they have been used as a model system to study electric-field-induced-motions by X-ray crystallography (Hekstra et al., 2016).

PDZ domains are peptide-recognition modules that bind to SLiMs, usually engaging with the extreme C-terminus of target proteins (Davey et al., 2012, Edwards et al., 2012, Ernst et al., 2009). PDZ domains directly interact in a conserved manner with up to 10 residues in a shallow binding cleft, the core of which is comprised of the βB strand and αB helix (Fig. 3, central panel) (Doyle et al., 1996). Some PDZ domains can recognize internal sequences, for example the Wnt signaling protein Dishevelled (Dvl)’s interaction with its target, the membrane-bound receptor Frizzled (Fz) (Nourry et al., 2003, Wong et al., 2003). Structural analysis revealed that Dvl is able to recognize internal ligand sequences, in addition to C-termini, due to inherent domain flexibility (Zhang et al., 2009). Internal binding motifs have been identified for a number of other PDZ domains, for example, in nNOS, PTP-BL, NHERF3, and the *Drosophila melanogaster* Par-6; this mode of binding may be particularly relevant in regulating GPCR signaling (Cha et al., 2017, Christopherson et al., 1999, Cuppen et al., 1998, Dunn and Ferguson, 2015, Harris et al., 2001, Lemaire and McPherson, 2006, London et al., 2004, Paasche et al., 2005, Penkert et al., 2004). In all cases, the internal motif forms a β-finger structure that forms an additional strand of the PDZ core antiparallel β-sheet, analogous to a C-terminal ligand (Paasche et al., 2005, Penkert et al., 2004). A structural example of this mode of binding can be seen in PDB accession code 1X8S of the *D. melanogaster* Par-6 PDZ domain bound to a peptide derived from PALS1 (Penkert et al., 2004). In addition, some PDZ domains have been shown to bind non-peptide ligands (e.g., phosphoinositides; see e.g., Ivarsson et al., 2013). Although potentially important physiologically, these non-canonical interactions are not a focus of this review.

A unique feature of crystallographic studies using PDZ domain constructs is that in certain crystal lattices, PDZ domains engage with the C-terminal tails of molecules related by crystallographic symmetry, allowing researchers to investigate target binding without addition of peptide ligands for co-crystallization (Elkins et al., 2010, Karthikeyan et al., 2001). Concurrently, this makes it difficult to identify ligand-bound PDZ domain structures by sequence-gazing or the number of protein chains in a PDB entry. We identified 170 peptide-bound domain structures (underlined in Table S2 are structures that include co-crystallized peptides plus selected structures with lattice contacts that mimic canonical interactions), or 33% of the unique domain structures. At least one peptide-bound structure is available for 57 of the 271 PDZ domains. At the same time, we acknowledge that this is likely to represent a lower bound on the number of PDZ domains with structurally resolved binding interactions due to the previous statement about lattice interactions. Of the identified structures, there are also several that contain small-molecule ligands, which are not highlighted explicitly in the table.

**Fig. 4:**
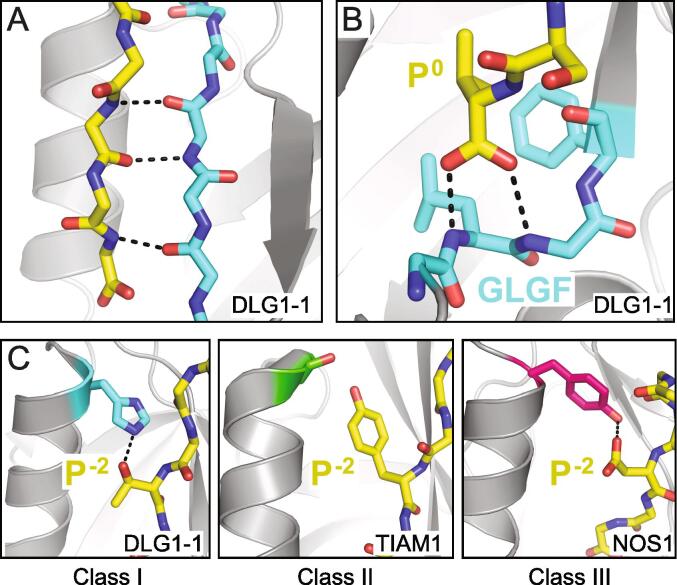
Conserved structural features of PDZ binding interactions. (A) The Dlg1 PDZ1 (DLG1-1) domain is in gray cartoon, with the exception of the βB strand (stick figure; cyan carbon atoms and heteroatoms colored by type). The APC peptide ligand is also shown as a stick figure (yellow carbon atoms). The peptide forms an additional strand of the core antiparallel β-sheet. Hydrogen bonds are shown as dashed lines, with distances (from bottom-to-top) of 2.7 Å, 3.0 Å, and 3.0 Å. (B) The carboxylate moiety of the P^0^ residue in APC interacts directly with the amides of the DLG1-1 “GLGF” motif (stick model; cyan carbon atoms). Hydrogen bond (dashed lines) are shown, with lengths from left-to-right of 2.9 Å and 3.3 Å. (C) Interactions of the PDZ domain with the P^−2^ residue historically determine binding class, and conserved features are shown for Classes I (DLG1-1, hydrogen bond, 3.0 Å), II (TIAM1, interacting residue in green sticks colored by heteroatom), and III (NOS, interacting residue in pink sticks colored by heteroatom; hydrogen bond, 2.9 Å) PDZ domains. PDB codes are: DLG1-1 (3RL7), TIAM1 (3KZE), NOS1 (1B8Q). For all, heteroatoms are colored by atom type (O = red and N = blue). (For interpretation of the references to colour in this figure legend, the reader is referred to the web version of this article.)

Relatively modest conformational changes are reported to be associated with peptide engagement. For example, there is an overall RMSD of 0.9 Å between the PSD-95 PDZ3 α-carbon positions in the first peptide-bound and the corresponding peptide-free crystal structure (Doyle et al., 1996). As mentioned previously, there is a standard conformation adopted by the C-terminal residues in most peptide ligands, in which the peptide forms an additional strand of the core β sheet (Fig. 4A). In addition, the main-chain terminal carboxylate group interacts with a conserved binding loop encoded by the “GLGF repeats” that Cho *et al*. had initially discovered as a major determinant of PDZ domain identity (Fig. 4B) (Cho et al., 1992, Ponting, 1997). Because of the structural conservation of both binding partners, the associated main-chain hydrogen bonds provide a shared baseline contribution to the thermodynamics of PDZ-peptide interactions.

### 1.3 Sequence motifs of PDZ ligands

In addition to main-chain interactions, PDZ domains also specifically recognize peptide side-chain residues, thereby imposing some level of target specificity. Originally, three distinct binding classes were defined for PDZ domain binding interactions: Class I PDZ domains recognize the motif sequence X-S/T-X-Φ (X = any amino acid, Φ = hydrophobic residues I, L, V, or F), Class II domains recognize X-Φ-X-Φ, and Class III domains recognize X-D/E-X-Φ (Ponting, 1997, Songyang et al., 1997, von Nandelstadh et al., 2009). In Class I binding interactions, the conserved histidine residue in the αB-1 position forms a hydrogen bond with the Ser/Thr residue in the P^−2^ position of the peptide, while in Class III, the P^−2^ Asp/Glu ligand residue interacts with a conserved tyrosine side chain at αB-1 (Fig. 4C) (e.g., Elkins et al., 2010, Songyang et al., 1997).

The similarities in structural fold, as well as the shallow nature of the PDZ binding cleft, result in a limited number of stereochemical restraints in peptide recognition and engagement, characteristic of SLiM binding (Davey et al., 2012). In general, PDZ binding interactions are often referred to as promiscuous, because in many cases, a number of PDZ domains can bind the same target, and *vice versa* (Gerek and Ozkan, 2010, Münz et al., 2012, Zhang et al., 2006). This likely reflects baseline affinity associated with the main-chain interactions described above. Despite these overlapping target specificities, the degenerate classifications described above are insufficient to circumscribe the interactome of each PDZ domain accurately.

A number of techniques have been used to identify more differentiated binding motifs for a number of PDZ domains, including many that are high-throughput: e.g., phage display, microarray, or peptide-array analysis (Duhoo et al., 2019, Luck et al., 2012, Stiffler et al., 2007, Stiffler et al., 2006, Tonikian et al., 2008). Here, a motif residue is defined by a preference for no more than four amino acids at a particular position relative to the C-termini of the ligands of a given domain. Notably, Tonikian *et al*. defined 16 distinct binding classes, dependent on the P^0^ and P^−2^ positions, as well as additional motif residues up to the P^−6^ position (Tonikian et al., 2008, Tonikian et al., 2007). These investigators successfully expressed and purified 88 human PDZ domains (as well as 57 from *C. elegans*), and determined binding motifs by testing high affinity interactions using phage display analysis. These extended motifs include PDZ domains Erbin, DLG1-3, INADL-2, and others (Tonikian et al., 2008). In 2014, this group structurally analyzed PDZ domains in all of the proposed binding classes (Ernst et al., 2014). A number of studies looking at PDZ binding selectivity and motif residues at positions outside of P^0^ and P^–2^ are reviewed in Luck *et al*. (Luck et al., 2012). Notably however, Tonikian *et al.*, as well as ourselves, continue to find a number of PDZ domains with a Class I-III degenerate motif, for example CAL, NHERF1, PTPN13-2, and SHANK3-1 (Amacher et al., 2014, Cushing et al., 2010, Tonikian et al., 2008, Vouilleme et al., 2010).

**Fig. 5:**
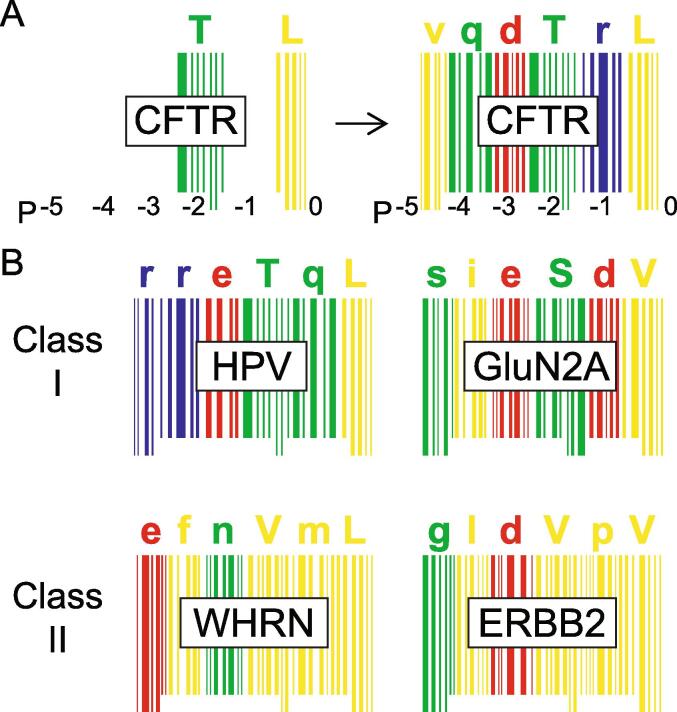
PDZ binding sequences as barcodes. (A) The C-terminal sequence of CFTR (VQDTRL) is shown as a barcode, meaning each residue is represented by a unique pattern of lines with varying thickness. Only motif residues are displayed in the left panel vs. all residues in the right panel. The colors correspond to the stereochemical nature of each residue: positively charged residues are blue, negatively charged residues are red, hydrophobic residues are yellow, and polar residues (including glycine) are green). Motif positions are shown as uppercase letters. (B) Additional common Class I (top panels) and Class II (bottom panels) PDZ binding targets are shown: HPV16 E6 (RRETQL), NMDA receptor subunit GluN2A (SIESDV), whirlin (WHRN, EFNVML), and receptor tyrosine-protein kinases erbB-2 (ERBB2, GLDVPV), are highlighted. The barcode analogy emphasizes that even similar sequences, particularly of the same binding class, can contain varied position-specific chemical and physical properties. (For interpretation of the references to colour in this figure legend, the reader is referred to the web version of this article.)

Overall, all residues that bind the PDZ domain binding cleft can potentially contribute to peptide selectivity and we can think of PDZ binding sequences as barcodes, in which the combination of each residue position encodes an overall sequence to be “read” by interacting PDZ domains (Fig. 5). Our lab previously defined the non-motif residue preferences within the peptide as *modulators*, in that these positional preferences can modulate affinity for peptide targets (Amacher et al., 2014). These preferences are important for sequence selectivity between two PDZ domains with degenerate or closely-related binding motifs (Vouilleme et al., 2010). It is important to remember that these extended motifs and modulator preferences are designed to identify the highest affinity binding sequences for a particular PDZ domain or set of domains. However, maximum affinity is not a requirement for endogenous interactions complicating the application of motif rules in the prediction of physiologically relevant PDZ interactomes. Furthermore, C-terminal sequences in the human proteome represent an extremely sparse sampling of all possible PDZ-binding sequences.

### 1.4 Structure-function relationships that control PDZ-Peptide affinity

**Fig. 6:**
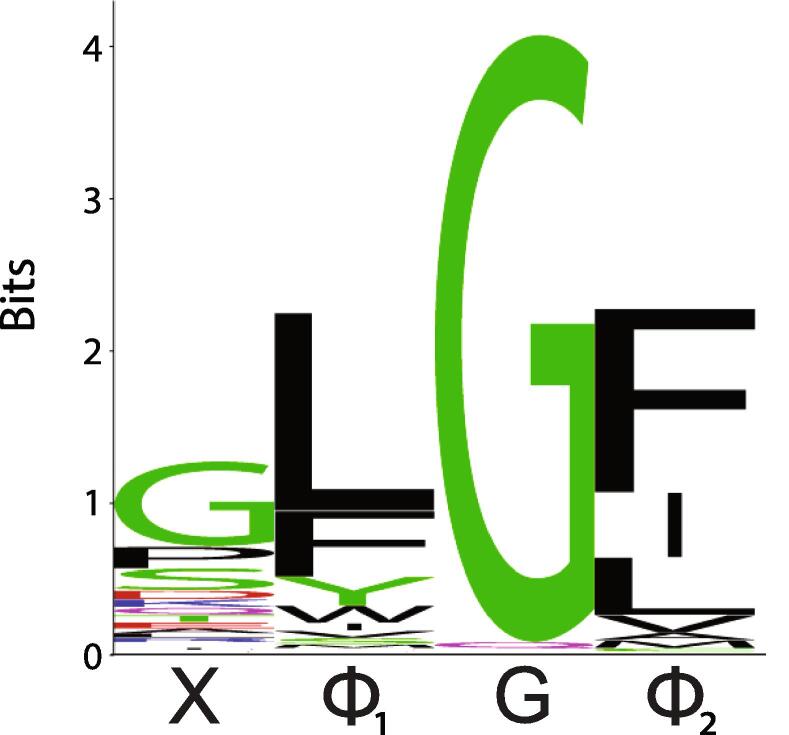
WebLogo analysis of carboxylate-binding loop sequences. Using structural information and sequence alignments, we identified the carboxylate-binding loop sequences of all known human PDZ domains, as reported in Table S3. WebLogo analysis reveals that the most common sequence is GLGF; however, there is substantial variability at the first, second, and fourth positions, which can affect peptide selectivity as previously reported (Amacher et al., 2013).

Because the overall domain structure is relatively independent of protein sequence variation, substitutions can preserve the fundamental ability of the domain to bind peptides, while altering its sequence specificity. In fact, at critical positions, even single substitutions can affect PDZ selectivity motifs. For example, the sequence of the carboxylate-binding loop has important implications on P^0^ selectivity. Although in the PDZ domains of PSD95 this sequence is GLGF, alignments soon identified variability in these residues (Ponting, 1997, Ponting and Phillips, 1995). In our hands, sequence alignments of PDZ domain sequences can misidentify the carboxylate-binding loop sequence. Those of known human PDZ domains are shown in Table S3, determined using available structural data in the case of ambiguities. An alignment using the WebLogo algorithm validates our updated XΦ_1_GΦ_2_ sequence motif (Fig. 6, Table S3).

The first residue in the motif, X, can represent any proteogenic amino acid, with one apparent exception. No human PDZ domain has a Trp residue in the first position. Analysis of the stereochemical basis of this absence, using structures of CAL (PDB ID: 4E34, X = Gly), Erbin (1N7T, X = Glu), HtrA1 (2JOA, X = Tyr), HtrA3 (2P3W, X = Phe), TIP-1 (4SFJ, X = Ile), Tiam1 (3KZE, X = Thr), and Scrib1 (2W4F, X = Glu), revealed that *in silico* mutagenesis resulted in clashes with either the peptide or residues in the βA–βB loop in all instances except HtrA1. These results support work from our group and others that investigated the stereochemical basis of P^0^ selectivity amongst PDZ domains that share a binding motif (Amacher et al., 2013, Appleton et al., 2006). The third residue is almost always a Gly, with three exceptions: CAR11 and CAR14 (Gln) and LNX1-1 (Ser) (Fig. 6, Table S3). We found that the identity of the Φ_1_ and Φ_2_ residues of the carboxylate-binding loop directly influence P^0^ residue selectivity (Amacher et al., 2013).

In addition, the ability to connect PDZ domain sequences directly to binding-motif preferences would facilitate computational modeling of the evolution of PDZ-mediated interaction networks. Previous work strongly indicates PDZ domains and their cellular targets co-evolved; where this has occurred, binding preferences are effectively hard-wired into the PDZ domain itself (Ernst et al., 2009, Kaneko et al., 2011, Kim et al., 2012, McLaughlin et al., 2012). Specifically, Ernst *et al.* found that by varying 10 binding site positions on the Erbin PDZ domain using phage display, they were able to generate variants with binding diversity comparable to that of the human PDZ family, including 7 specificity clusters not yet found in nature (Ernst et al., 2009). In addition, McLaughlin *et al*. used a bacterial two-hybrid (B2H) system to mutate each residue in the PSD95-PDZ3 domain to each of the other proteogenic amino acids and then to investigate quantitatively how these variants affect ligand binding. They report that 20 out of 81 total sites on the PSD-95 PDZ3 domain functionally affect binding *via* multiple cooperation networks between residues (McLaughlin et al., 2012). On a PDZome-wide scale, the holdup assay provides a high-throughput technique to quantitate peptide binding to almost the full complement of human PDZ domains. This experimental assay uses microfluidic capillary electrophoresis to measure binding affinities of 266 recombinantly produced human PDZ domains, as well as 87 tandem domains, with peptide-coated resins, measuring up to 1000 binding affinities per day (Duhoo et al., 2019, Vincentelli et al., 2015).

In another set of studies, site-directed mutagenesis of PDZ domains was clearly shown to influence binding specificity, and the prediction of the interactomes of multiply mutated Erbin PDZ domains was the focus of a blind prediction challenge in the DREAM4 (Dialogue for Reverse Engineering Assessments and Methods) Consortium, which provided rigorous benchmarks for computational methods (e.g., refs. Smith and Kortemme, 2010, Zaslavsky et al., 2010). Indeed, one of the hallmarks of organismal complexity is the expansion of the number of PDZ domains and the rewiring of their interactions (Chimura et al., 2011, Kim et al., 2012, Sakarya et al., 2010). Underscoring the target flexibility achievable by residue substitutions in PDZ domains, Teyra *et al*. recently found that only 3 mutations were sufficient to describe the specificity-switching between Erbin and Pdlim4 PDZ domains, suggesting that only extremely short evolutionary pathways were necessary to introduce complexity into this protein family (Teyra et al., 2019).

### 1.5 The Variable world of PDZ binding affinities

**Fig. 7:**
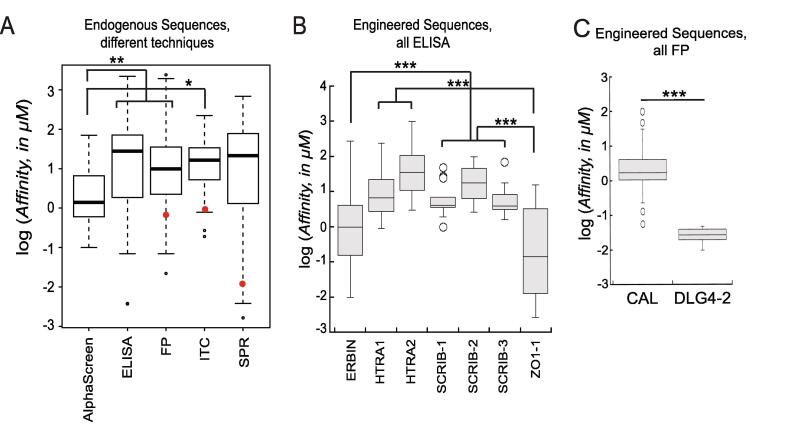
Binding affinities analyzed by experimental technique (A) and domain identity (B–C). (A) We compiled a sampling of previously reported binding affinities for sequences derived from endogenous binding partners (Table S4), measured using a variety of techniques (e.g., FP, SPR, ITC). Statistical comparisons reveal a difference in affinity estimates associated with AlphaScreen compared to the other techniques. Avidity effects can affect measurements using non-solution-based techniques, such as AlphaScreen, but also ELISA and SPR, depending on how the experiment is performed. For example, affinity values were estimated in parallel using different techniques for NHERF1-1 binding to CFTR and are marked with red circles on the FP (*K*_D_ = 597 nM), ITC (*K*_D_ = 787 nM), and SPR (*K*_D_ = 10.3 nM) bars. (B, C) Experimental binding affinities were also compiled for high affinity engineered interactors (Table S6). (B) Comparisons among sequences developed using phage display, reveal that these proteins bind to their optimized sequences with a different range of affinities. (C) Experimental binding affinities of high affinity interactors developed using fluorescence polarization (FP) analysis for the CAL and Dlg4-2 PDZ domains, also reveal a statistically significant difference in affinities. For all, * p ≤ 0.05 or ** p ≤ 0.01 by linear test (A) or *** p ≤ 0.005 by one-way ANOVA using Tukey’s HSD post-hoc test (B–C).

The published literature includes hundreds of papers that report binding affinities for PDZ domains and their target sequences. Likely this is due, at least in part, to the relative ease of expressing and purifying many of these small modular domains, as well as the ability to synthesize binding peptides. The most common techniques used to determine PDZ binding affinities are fluorescence polarization (FP) and surface plasmon resonance (SPR) (Frostell et al., 2013, Rossi and Taylor, 2011). Importantly, FP is a solution-based technique that utilizes a fluorescent tag, which can influence binding, while SPR requires one partner of the complex, either the peptide or PDZ domain, to be fixed to a surface. As a result, SPR may be subject to avidity effects for multivalent complexes, and many early measurements reported inaccurate values of PDZ domain binding, generating artificial values in the low nanomolar range (Cushing et al., 2008). Previous work from our lab obtained concordant values for the affinity of NHERF1-1 for a CFTR C-terminal decamer (TEEEVQDTRL) using FP (*K*_I_ = 597 nM) and isothermal titration calorimetry (ITC) (*K*_D_ = 787 nM), whereas SPR yielded a much higher-affinity interaction (*K*_D_ = 10.3 nM), consistent with previously published work (see asterisks in Fig. 7A). This discrepancy most likely reflects SPR surface avidity effects and appears to be particularly problematic when the peptide is immobilized as the surface ligand, rather than the protein domain (Cushing et al., 2008). In addition to these three techniques, AlphaScreen proximity assays, enzyme-linked immunosorbent assays (ELISA), nuclear magnetic resonance (NMR), Trp fluorescence, and additional binding assays, for example hold-up and Coomassie-stained gel-based assays, have all been used to measure binding affinities.

We collated PDZ binding affinity values from over 80 studies using multiple distinct binding techniques, and confirmed that PDZ domain binding affinities fall in the expected range for SLiM interactions – centered in the micromolar range, with median values of 1–30 μM (Table S4) (Davey et al., 2012). However, the values collectively span a very wide range, from the high-nanomolar (e.g., TIP-1:β-catenin at ∼300 nM or NHERF2:CFTR at ∼150 nM) to the high-micromolar range (e.g., CAL:CFTR at ∼400 μM), even for the same target peptide (e.g., CFTR) (Amacher et al., 2013, Cushing et al., 2010, Maki et al., 2007, Zhang et al., 2008). An analysis of variance (ANOVA) with a post-hoc Tukey’s HSD (honestly significant difference) test was used to assess differences based on the measurement technique used, for the five most common techniques (AlphaScreen, ELISA, FP, ITC, and SPR) (Table S4, Fig. 7A). A total of 365 numerical affinity values were used in the comparison. Assuming that the listed values are representative, pairwise comparisons of the five most commonly used techniques did not show differences that were significant at the *p* < 0.05 level. However, the AlphaScreen data are skewed noticeably lower than the other four techniques (Fig. 7A), and in our unbiased comparison, approached this significance threshold in comparison to ELISA and FP affinity values.

From a mechanistic perspective, the AlphaScreen assay detects the physical proximity of ‘donor’ and ‘acceptor’ beads that are each coated with one binding partner or the other. As a result, both interacting entities are typically multivalent. In comparison, in ELISA and SPR assays, only one binding partner is immobilized on a multivalent surface, while the other is in solution, and in FP and ITC assays, neither partner is immobilized. To test the specific hypothesis that AlphaScreen estimates (often IC_50_ or EC_50_ values) reflect avidity effects more strongly than the other techniques, we utilized a linear model, and found significant differences for values determined using AlphaScreen, as compared to the other techniques, including ELISA (*p* = 0.007), FP (*p* = 0.006), ITC (*p* = 0.037), and SPR (*p* = 0.05).

We also explored differences between the sets of affinities associated with distinct PDZ domains. Given the methodological differences described above, we performed a two-way ANOVA, taking both domain identity and methodological variation into account, and using a post-hoc Tukey’s HSD test in order to evaluate the hypothesis that higher affinity peptides may be available for some domains than for others. Assuming that the selected affinities are representative, of 1540 pairwise comparisons between affinity estimates for 56 single domains, 94 showed *p*_adj_ values <0.05. 95% confidence intervals are shown in Table S5 for the differences in estimated affinities for each of these comparisons. We then tabulated 200 affinities of engineered sequences for 12 different PDZ domains, giving greater depth of coverage to a smaller number of domains, (Table S6). We repeated the analysis and found 22 of the possible 66 pairwise comparisons of domain affinity ranges exhibited statistically significant differences, including 5 pairings seen using endogenous target affinities, and 17 new pairings (Table S7). For ease of visualization, we also performed a simplified analysis comparing peptide affinities for seven different domains, all obtained using ELISA-based assays (Fig. 7B). A one-way ANOVA with Tukey’s HSD test reveals multiple differences. For example, while the Erbin PDZ domain bound a number of peptides with affinities as low as 10 nM, the highest affinity peptide for the SCRIB PDZ3 domain was 1600 nM (*p_adj_* < 0.005). We similarly find a statistically significant difference between CAL and PSD-95 PDZ2, both measured by FP (Fig. 7C). All of the phage display-derived experiments, measured with ELISA, were from the same group, whereas different labs, including ours, engineered the CAL and PSD-95 PDZ2 peptides for FP measurements (Amacher et al., 2013, Roberts et al., 2012, Runyon et al., 2007, Skelton et al., 2003, Tonikian et al., 2007, Vouilleme et al., 2010, You et al., 2006). Interestingly, even better targeting of PDZ proteins can be achieved by leveraging avidity effects and the presence of multiple domains, for example, Bach *et al.* reveal 10-100x stronger inhibitor binding to PSD-95 PDZs 1&2 by using a dimeric peptide, then when targeting each PDZ domain individually (Bach et al., 2012).

Despite the relatively high affinity of Erbin for engineered sequences, only 2 peptides that match endogenous cellular target sequences reveal binding affinities in the nanomolar range (Table S4) (Jaulin-Bastard et al., 2001, Laura et al., 2002, Wiedemann et al., 2004, Zhang et al., 2006). For CAL, there are a number of peptide sequences that bind in the single micromolar range; however, the highest affinity endogenous sequences bind with ≥10-fold weaker affinity (Amacher et al., 2013, Cushing et al., 2008, Roberts et al., 2012). The ability of PDZ domains to transiently interact with non-optimal sequences makes interactome studies challenging. Furthermore, this characteristic is exploited by invading pathogens, specifically viruses.

### 1.6 Viruses target PDZ domain networks

Viral proteins are known to affect two major host mechanisms via PDZ domain interactions: 1. Disruption of tight junction formation in epithelial cells, and 2. Blocking apoptosis of cellular components, leading to uncontrolled cell growth (Javier and Rice, 2011, Lee and Laimins, 2004). In the late 1990s, researchers identified three viral oncoproteins (adenovirus type 9 E4-ORF1, human T-lymphotropic virus type 1 Tax, and high-risk human papillomavirus (HPV) E6) that contain C-terminal PDZ domain binding motifs. They also showed that these proteins target PDZ domains, such as the tumor suppressor proteins Dlg and hScrib, leading to proteasome-mediated degradation (Gardiol et al., 1999, Javier and Rice, 2011, Nakagawa and Huibregtse, 2000, Pim et al., 2012).

The list of PDZ domains targeted by the HPV E6 proteins, specifically those of the cancer-associated HPV-16 and HPV-18 strains, now includes MAGI-1, MAGI-2, MAGI-3, PSD95, GIPC, MPDZ, PATJ, PTPN3, PTPN13, CAL, NHERF1, TIP-1, and others (Accardi et al., 2011, Belotti et al., 2013, Fournane et al., 2011, Jeong et al., 2007, Mischo et al., 2013, Oliver et al., 2011, Pim et al., 2012, Tungteakkhun and Duerksen-Hughes, 2008, White et al., 2012, Zhang et al., 2007). The number of known viruses whose proteins target PDZ domains has also expanded dramatically to include: influenza A, rabies virus, human immunodeficiency virus (HIV), and severe acute respiratory syndrome (SARS) virus (James and Roberts, 2016, Javier and Rice, 2011, Kim et al., 2014, Shepardson et al., 2019). For example, a number of HIV proteins are known to interact with PDZ domains, including HIV-1 glycoprotein 120, which is derived from the HIV-1 glycoprotein 160 precursor (James and Roberts, 2016). Specifically, the last 5 residues of UniProt codes ENV_HV1AN, with sequence ERSLL, and ENV_HV2D2, with sequence ELTLL, differ from that of known PDZ-binding protein Myosin 15a, with sequence EITLL, by ≤2 residues, and maintain canonical PDZ binding motifs (Belyantseva et al., 2005). For reference, there are two recent reviews of known viral protein-PDZ interactions, including discussions of how viruses exploit PDZ targeting to evade the immune system (Gutiérrez-González and Santos-Mendoza, 2019, James and Roberts, 2016).

**Fig. 8:**
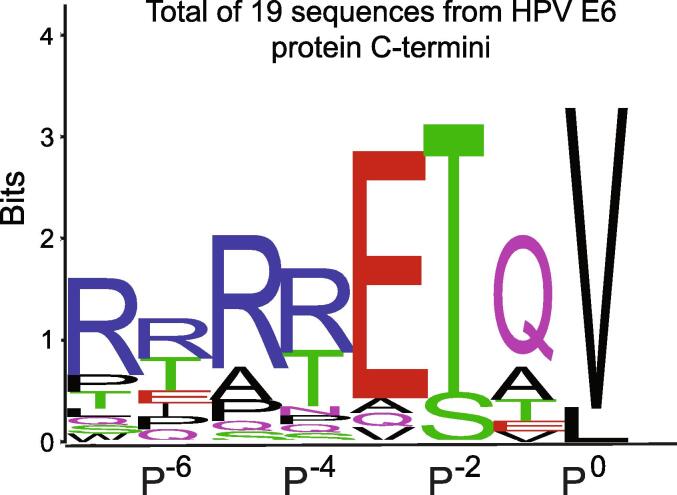
WebLogo analysis of PDZ motif-satisfying HPV E6 sequences. A WebLogo analysis of the C-terminal sequences of 19 E6 oncoproteins from various HPV strains reveals that the most common PDZ-targeting sequence motif in these proteins is RRRRETQV.

Amongst the HPV strains, 13–18 are termed high-risk for developing cancer (with the highest risk attributed to the HPV-16 and HPV-18 strains), all of which have PDZ binding motif-satisfying E6 proteins (Ault, 2007). The C-terminal sequences of 19 of the 65 reviewed E6 protein sequences listed in UniProt reveal canonical binding motifs. WebLogo analysis of these sequences confirms that the most prevalent residues match the HPV-18 E6 C-terminal sequence (RRETQV) (Fig. 8). Furthermore, this viral sequence matches the consensus sequence for protein kinase A (PKA), X-R-R-X-S/T-X. Phosphorylation of the P^−2^ Thr residue in HPV-18 has been shown to inhibit E6-induced degradation of Dlg in HEK293 cells, as well as decrease cellular growth of primary keratinocytes (Delury et al., 2013, Kühne et al., 2000). It is interesting that the viral sequence has not evolved to evade this host-mediated inhibition by PKA. It suggests that in addition to motif-satisfying amino acids, the Arg residues may be important for PDZ domain targeting.

### 1.7 Therapeutically targeting PDZ domains to combat human disease

**Fig. 9:**
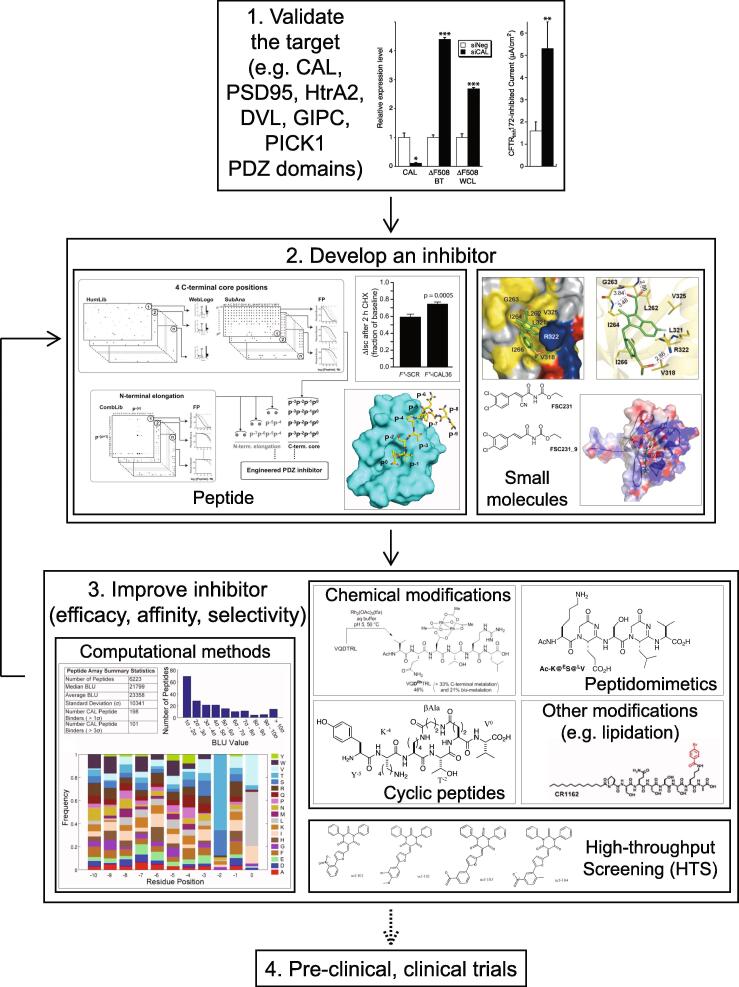
Drug development pipeline for PDZ domain inhibitors. A general pipeline is shown for target validation, inhibitor development, and inhibitor refinement for select PDZ domain inhibitors, as assessed by a literature review. Figures have been adapted from references as follows: top panel (Wolde et al., 2007); middle panel (Amacher et al., 2013, Cushing et al., 2010, Lee et al., 2009, Thorsen et al., 2010, Vouilleme et al., 2010); bottom panel (Cilenti et al., 2003, Hammond et al., 2006, Kundu et al., 2012, Patra et al., 2012, Piserchio et al., 2004, Roberts et al., 2012).

Understanding how PDZ domains recognize their targets and what determines binding preferences is extremely useful in engineering specific PDZ inhibitors, because each peptide-binding cleft can be treated as a pharmacophore. The interacting surface for each position is referred to as a site or socket, for example, S^0^ interacts with residue P^0^, S^−1^ with P^−1^, etc. (Amacher et al., 2014, Boucherle et al., 2011, Madsen et al., 2005). To date, inhibitor development has followed a general pipeline, as summarized in Fig. 9. Initially, a target is validated *via* cell-based experiments (e.g., Cushing et al., 2010, Wolde et al., 2007). For example, in the disease cystic fibrosis, levels of CFTR are reduced at the epithelial cell surface leading to improper hydration of the airway surface liquid and a buildup of mucus and bacterial infection in the patient (Rogan et al., 2011). The CFTR-associated ligand CAL has a PDZ domain that binds the C-terminus of CFTR, triggering its lysosomal degradation, and knockdown of CAL using siRNA reveals a robust increase in cell surface CFTR expression, as well as an increase in CFTR-mediated Cl^−^ currents across epithelial cell monolayers (Cushing et al., 2010). In another example, knockdown of Dvl using siRNA, as well as expression of Dvl mutants, was shown to inhibit tumor growth, by disrupting the Wnt signaling pathway and transcriptional activation of β-catenin, in addition to other targets (Uematsu et al., 2003a, Uematsu et al., 2003b) (Fig. 9, top panel).

Following target validation, inhibitors are designed using either a peptide- or small molecule-based scaffold, and screened via a high throughput method, e.g., peptide array, phage display or high throughput screens (HTS) (Boisguerin et al., 2007, Chen et al., 2007, Cilenti et al., 2003, Cushing et al., 2010, Hammond et al., 2006, Kaneko et al., 2011, Thorsen et al., 2011, Vouilleme et al., 2010, Wiedemann et al., 2004, Zhang et al., 2009). As mentioned previously, leveraging avidity effects with dimeric or trimeric inhibitors show increased affinity, and efficacy, against PDZ domain targets (Bach et al., 2012, Nissen et al., 2015). This phase involves a number of iterative rounds, optimizing selectivity and affinity for the PDZ domain of interest (Fig. 9, middle panel). As with other drugs, cellular delivery is a significant hurdle. In response, cell-penetrating peptides or delivery agents to improve methods are being actively developed (Patra et al., 2012, Piserchio et al., 2004, Tao and Johns, 2010). The advancements of PDZ domain therapeutics additionally depend on the capability to dissect the interacting networks wherein these proteins function in the cell. Therapeutic design is progressing, for example, a peptide-based PSD-95 PDZ2 inhibitor, targeting stroke and first developed in 2002, called “NA-1” completed Phase III clinical trials in November 2019 (Aarts et al., 2002, Ballarin and Tymianski, 2018, Christensen et al., 2019). A number of other inhibitors are at various phases of development or in preclinical studies, as comprehensively reviewed in (Christensen et al., 2019).

### 1.8 There is no “Ile” in “Thr-Glu-Ala-Met”, or how all of the residues contribute to the interaction

Over the next two sections of this review, we will shift our focus to the regulation of PDZ binding interactions. Outside of the peptide-binding cleft, there are a number of residues that influence target recognition. Indeed, this is due to extensive allosteric networks in PDZ domains. Lockless and Ranganathan first measured energetic couplings that included the Class I αB-1 His residue of PSD-95 PDZ3, residues throughout the core of the protein, and the αA helix, in 1999 (Lockless and Ranganathan, 1999). This type of pathway is defined as a sector, or a “sparse network of physically contiguous and coevolving amino acids” (Reynolds et al., 2011). After mutating all 39 surface-exposed residues on PSD-95 PDZ3 to all other proteogenic amino acids, 11 residues were found to have significant effects on CRIPT ligand binding, with 10 of these being sector connected (Reynolds et al., 2011). Overall, it is clear that allosteric effects are major determinants of PDZ binding interactions (Gautier et al., 2018, Karlsson et al., 2016, Kumawat and Chakrabarty, 2019, Kumawat and Chakrabarty, 2017, Murciano-Calles et al., 2014, Raman et al., 2016). For a broad discussion of inter- and intracellular communication pathways in protein signaling domains, including PDZ domains, see a number of reviews (Gautier et al., 2018, Karlsson et al., 2016, Smock and Gierasch, 2009).

**Fig. 10:**
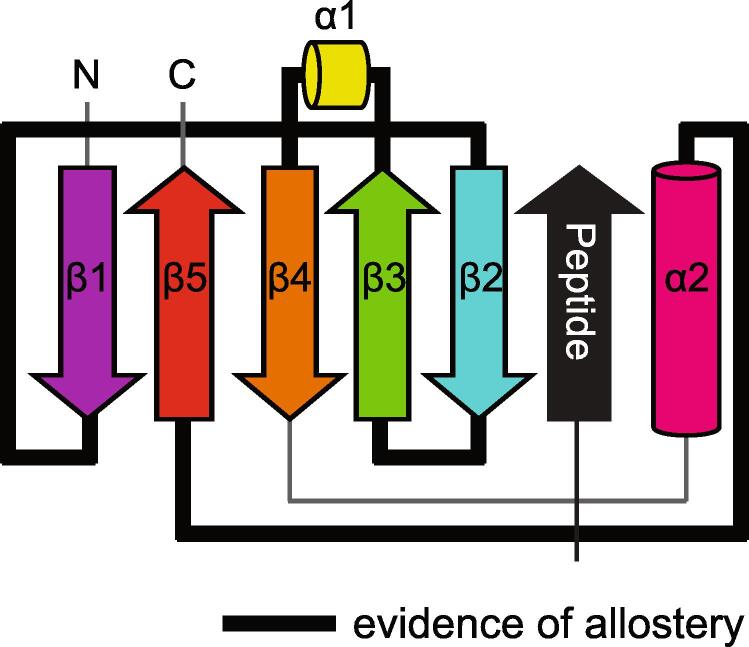
Reported regions of allostery in a PDZ domain. A two-dimensional schematic of the common PDZ domain fold is shown. The loops that have shown evidence of allostery are highlighted in bold. In addition, residues throughout the PDZ domain are implicated in long-range intradomain allosteric networks. This schematic was first published in (Valgardson et al., 2019).

Additional studies using NMR, kinetic experiments, and extensive mutagenesis on PSD-95 PDZ3, Par-6, PTP-BL PDZ2, and hPTP1E PDZ2 identify residues in the βA-βB and βB-βC loops as modulators of ligand binding (highlighted in Fig. 10) (Fuentes et al., 2004, Gianni et al., 2011, Peterson et al., 2004, Whitney et al., 2011). There is also evidence of an effect of residues in the βD-βE loop, αA-βD loop, and the αC helix of PSD95 PDZ3, suggesting that almost all of the loops and secondary structure elements of a PDZ domain can contribute to binding selectivity (Fig. 10) (Feng et al., 2002, Gianni et al., 2011, Petit et al., 2009). Mutation of these sites can reduce ligand affinity up to 21-fold, suggesting additional target binding surfaces for therapeutic development. Indeed, a covalent allosteric inhibitor weakens peptide-binding affinity of the CAL PDZ domain via modification of a Cys residue located outside of the peptide-binding cleft (Zhao et al., 2018).

Algorithms that investigate PDZ interactions have greatly improved in their ability to parameterize PDZ binding and predict relatively high affinity interactions *in silico*. In general, we and others are attempting to use available sequence and structural information to predict binding affinities with the goal of identifying cellular interaction networks (e.g., refs. Gerek et al., 2009, Gerek and Ozkan, 2010, Gfeller et al., 2011, Holt et al., 2019, Landgraf et al., 2004, te Velthuis et al., 2011, Tian et al., 2011, Valgardson et al., 2019). Understanding structural flexibility is important to these studies (Roberts et al., 2012, Thomas et al., 2009). However, these efforts are complicated by the tight regulation of residue accessibility and domain localization on both sides of the interaction, as well as by post-translational modifications or lipid binding.

### 1.9 Regulation of PDZ domains and their targets

An important regulatory mechanism of many PDZ domains is their association with the plasma membrane. Some of the most well studied PDZ domains are those of the membrane-associated guanylate kinase (MAGUK) protein family. Lipid-binding characteristics of PDZ domains have long been studied (e.g., refs. Das et al., 2003, Kachel et al., 2003, Wu et al., 2007, Zimmermann et al., 2002). A computational and experimental study by Chen *et al*. suggests that approximately 30% of human PDZ domains bind lipids, with 2 distinct modes of binding that can affect ligand affinity, either positively or negatively (Chen et al., 2012). These interactions are selective, and dependent on lipid head group. Of the interactions measured experimentally, most are in the nanomolar range (the authors used 1 μM as a cutoff to determine “binding” *versus* “non-binding”), and the tightest interaction was for NHERF1 PDZ1, at 24 nM (Chen et al., 2012). Membrane association localizes PDZ domain-containing proteins into distinct compartments.

Additional methods of regulation include a number of post-translational modifications that affect PDZ domains (e.g., refs. Chung et al., 2002, Fukata and Fukata, 2010). Here, we will focus on phosphorylation, which is the most well-studied and which often disrupts PDZ domain:target interactions both within and outside the peptide-binding cleft. A group of papers published in 2002 identified phosphorylation of the C-termini of potassium channel Kir5.1, β1-adrenergic receptors, and stargazin as a mechanism for disrupting PDZ domain recognition (Chetkovich et al., 2002, Choi et al., 2002, Hu et al., 2002, Kim and Sheng, 2004, Tanemoto et al., 2002). Additional examples of C-terminal sequence modifications that regulate PDZ domain binding include the phosphorylation of Ser880 in the AMPA receptor subunit GluA2 by protein kinase C (PKC), of potassium channel Kir2.3 by protein kinase A (PKA), and of the β2 adrenergic receptor (β_2_AR) by G protein-coupled receptor kinase 5, GRK5 (Cao et al., 1999, Chung et al., 2000, Cohen et al., 1996, Leonoudakis et al., 2001, Matsuda et al., 1999). These phosphorylation events directly affect binding to PDZ domains in GRIP, PSD-95, Dlg1, and NHERF1 (Kulangara et al., 2007, Narayan et al., 2009, Nelson et al., 2013, Voltz et al., 2007, Wang et al., 2012). However, these receptors are targets of additional PDZ domains, such as SNX27, TIP-1, PICK1, LIN7A-C, and CASK, and these interactions may also be affected (Perez et al., 2001, UniProt Consortium, 2019).

Recent studies investigating the cellular impact of PDZ ligand phosphorylation reveal that these post translational modifications have a dramatic impact on the global landscape of PDZ binding interactions (Gógl et al., 2019, Sundell et al., 2018). Specifically, proteomic analysis of binding interactions to either a phosphorylated or unphosphorylated version of the C-terminal peptide of ribosomal S6 kinase 1 (RSK1), which can be phosphorylated in response to epidermal growth factor (EGFR) signaling, revealed both enhanced and weakened affinity in the interactome of RSK1:PDZ domain interactions (Gógl et al., 2019). In addition, phage display analysis of the Scribble and DLG1 PDZ domains with unphosphorylated or phosphomimetic peptides of endogenous PDZ ligand sequences again suggest that phosphorylation is a powerful regulatory mechanism for altering PDZ binding and affinity of cellular targets (Sundell et al., 2018).

These examples all highlight Ser/Thr phosphorylation at the P^−2^ position of Class I PDZ binding motifs. Another residue that is often phosphorylated is tyrosine. In 2013, Liu *et al*. crystallized and determined the structures of the Tiam1 PDZ domain bound to the last 8 residues of syndecan1 (SDC1, sequence TKQEEFYA) as well as a phospho-SDC1 peptide (pSDC1), with a phosphorylated P^−1^ Tyr (pY) (Liu et al., 2013). Another group identified an ∼3-fold decrease in the Afadin PDZ:Jagged-1 interaction, in the presence of P^−2^ Tyr phosphorylation (Popovic et al., 2011).

Phosphorylation of the PDZ domain itself can also regulate PDZ domain interactions. There are a number of examples of these types of events, either via direct binding or allosteric mechanisms. One of the earliest examples is Ca^2+^/calmodulin-dependent protein kinase (CamKII)-dependent phosphorylation of Dlg1 at Ser232, the residue immediately preceding the carboxylate-binding loop sequence, disrupting its interaction with the NMDA receptor subunit GluN2A both *in vitro* and in Cos7 cells (Gardoni et al., 2003). Notably, this Ser residue is conserved in the first PDZ domain of the other Dlg proteins, including PSD-95. In addition, phosphorylation of Tyr397 in PSD-95 PDZ3 allosterically regulates its conformation and interaction with its own SH3 domain (Zhang et al., 2011a, Zhang et al., 2011b). More recently, site-specific phosphorylation of PSD-95 revealed that phosphorylation of Y397 in PSD-95 resulted in a significant increase in affinity for stargazin (Pedersen et al., 2017). Additional PDZ domains regulated *via* phosphorylation mechanisms include NHERF1 and PTEN (Adey et al., 2000, Hall et al., 1999).

Phosphorylation sites can also be engineered into the PDZ domain to affect ligand binding. An example is the design of a phosphorylatable Erbin PDZ domain by Smith *et al*. (Smith and Kortemme, 2010). This group successfully engineered cAMP dependent protein kinase (PKA) recognition sites into the Erbin PDZ domain sequence, verified by mass spectrometry following *in vitro* PKA phosphorylation. In all 8 positions they tested, serine phosphorylation resulted in a decrease of binding affinity to synthetic peptides (Smith and Kortemme, 2010). Importantly, a number of these phosphorylation sites were outside the peptide-binding cleft, again highlighting the ability of sites throughout the PDZ domain to affect ligand recognition *via* allosteric networks.

### 1.10 It takes a village to build a protein complex

PDZ domain or target regulation by phosphorylation requires additional interactions with kinases and/or phosphatases. So far, this review has focused on characteristics of individual PDZ domains, but PDZ-mediated multivalent interactions depend on molecular recognition beyond an isolated binding event between a PDZ domain and its target. Some PDZ domain-containing proteins, like TIP-1 (TX1B3) are comprised almost entirely of a single PDZ domain; however, this is the exception not the norm. A more common feature of PDZ domain-containing proteins is the presence of multiple interaction domains, including additional PDZ domains (Fig. 2, Table S1) (Kelil and Michnick, 2019, Kim and Sheng, 2004, Lee and Zheng, 2010, Nourry et al., 2003, Ye and Zhang, 2013). The coordination of these modules in multivalent interactions is important for molecular recognition, directly regulating the local concentration of components (Albertazzi et al., 2013). Multivalency in signaling proteins also creates macromolecular complexes with different physical and chemical properties than those at the single-molecule scale (Kelil and Michnick, 2019, Li et al., 2012).

Epithelial cells provide a good example. The PDZ protein connector enhancer of kinase suppressor of Ras isoform 3 (CNKR3) acts as an important regulatory signal by scaffolding the epithelial Na^+^ channel (ENaC) which is crucial for proper Na^+^/K^+^ balance. CNKR3 also scaffolds the serine-threonine kinase SGK1 and ENaC inhibitor Nedd4-2, in a >1 MDa complex which plays an important role in Na^+^ homeostasis (Soundararajan et al., 2012). In the post-synaptic density of neurons, perhaps the most well-studied example, the PDZ domain-containing Shank proteins act as molecular bridges between the metabotropic glutamate receptors (mGluRs) and the actin cytoskeleton (Kim and Sheng, 2004, Sheng and Kim, 2000, Vessey and Karra, 2007). For a review on the role of PDZ domain proteins in neuronal synapses, specifically PSD-95, see previously published reviews (Kim and Sheng, 2004, Manjunath et al., 2018).

There are also complexes that consist of multiple PDZ domain-containing proteins. MPP7, for example, forms a tripartite complex with discs large homolog 1 (DLG1) and any of the lin-7 homolog family members (Lin7A-C), drawing together a total of 5 PDZ domains that are important for the proper localization of cell–cell junction components (Bohl et al., 2007). Due to the identification of individual binding motifs, we know that in this complex, DLG1 (specifically, DLG1-3) and Lin7A share almost identical binding motifs, dependent on 6 residues (Bohl et al., 2007, Tonikian et al., 2008). Therefore, by forming this complex, there are additional PDZ domains available to bind similar targets, instead of those solely in DLG1.

Our sequence identity tree of PDZ domain-containing proteins clusters proteins by auxiliary domains, since we are aligning the entire protein sequence, including non-PDZ domains (Fig. 1A). We defined a *node* as the closest branch point to the central arc of the rooted tree, and distinctly colored the PDZ domain containing-proteins of each node. Our tree agrees with previously reported PDZ families of many members, for example, those of the MAGUK family, characterized by a PDZ-SH3-guanylate kinase “supradomain” architecture (J. Zhang et al., 2011a, Zhang et al., 2011b). We also identify PDZ proteins that do not share strong similarity to other proteins, for example ARHGB and NOS1. The non-PDZ domains in these proteins are listed in Table S1. Overall, the most common of the approximately 60 associated domains among PDZ domain-containing proteins are the SRC Homology 3 (SH3), guanylate kinase, LIM, and L27 domains, which are all signaling or scaffolding domains. Interaction cooperativity among domains of a complex or single protein is not well understood, and represents the next frontier in understanding signaling pathways at both the molecular and systems levels.

## 2 Concluding remarks

PDZ domains and PDZ domain-containing proteins are as diverse as the targets they recognize. This review summarizes work on the biochemical and biophysical characterization of these interaction modules, specifically focusing on available sequence and structural information and binding data. Hopefully, these analyses provide insight into common traits among these protein modules, but also, elucidate specific examples that reveal the unique character of each PDZ domain. Thus, while future work needs to focus on discovery at the whole-network level (e.g., using the large repositories of data on phosphorylation events in a variety of cell types and species to isolate phosphorylated sites in PDZ domains and target proteins), we also need in-depth work on less well studied PDZ domains (e.g., data in Beltrao et al., 2012, Gnad et al., 2011, Gnad et al., 2007, Kettenbach et al., 2011).

Fortunately, there are a number of analogies to aid future work. Kinases, for example, share many SLiM engagement characteristics with PDZ domains. These include the recognition of up to 9 residues in their catalytic site and the use of similar stereochemical and electrostatic mechanisms as PDZ domains (Cantor et al., 2018, Shah et al., 2018, Shah et al., 2016, Ubersax and Ferrell, 2007). This results in binding motifs of vastly different numbers of residues. Kinases also utilize localization, local concentration, and auxiliary domains to engage their substrates (Ubersax and Ferrell, 2007). Likely, PDZ domains are recognizing targets in a similar manner.

PDZ domain target recognition is also very similar to that of SH2 domains, which recognize phosphotyrosine residues downstream of tyrosine kinases. SH2 domain binding motifs are also insufficient to describe these SLiM interactions, and a deeper understanding of modulator-like preferences, both positive and negative, is needed to describe the interactome of each domain (Liu et al., 2010). These are only two examples, and there are a number of other SLiM binding domains to be considered, including WW, SH3, PTB, and PH domains.

As the basis of future work, we need to start thinking more holistically, treating a combination of signaling domains as one functional entity. There are groups who are doing this, either by cataloguing the interactomes of promiscuous modular domains, then diagramming interaction networks, or by looking at different size scales in the cell that result from macromolecular complexes (e.g., Cumberworth et al., 2013, Li et al., 2012). In order to organize this information, we need to apply constraints (e.g., binding affinity and structural data) from each domain on the individual level. As our computing power and technology improves, we will be able to combine target and localization data on all of these domains simultaneously to simulate cellular processes, and gain a systems level understanding, as previously suggested (Fraser et al., 2013). This will ultimately allow us to move towards a global understanding of the interaction networks that govern the behavior of our cells.

## Declaration of Competing Interest

The authors declare that they have no known competing financial interests or personal relationships that could have appeared to influence the work reported in this paper.
