# Supplementary material for: Specificity in PDZ-peptide interaction networks: Computational analysis and review
Source: J Struct Biol X. 2020 Mar 7;4:100022. doi: 10.1016/j.yjsbx.2020.100022 (PMC7138185; doi:10.1016/j.yjsbx.2020.100022)
Supplement: Supplementary data 7 [file mmc7.pdf]

Table S7: Statistically significant pairwise affinity differences between PDZ domains binding engineered targets.

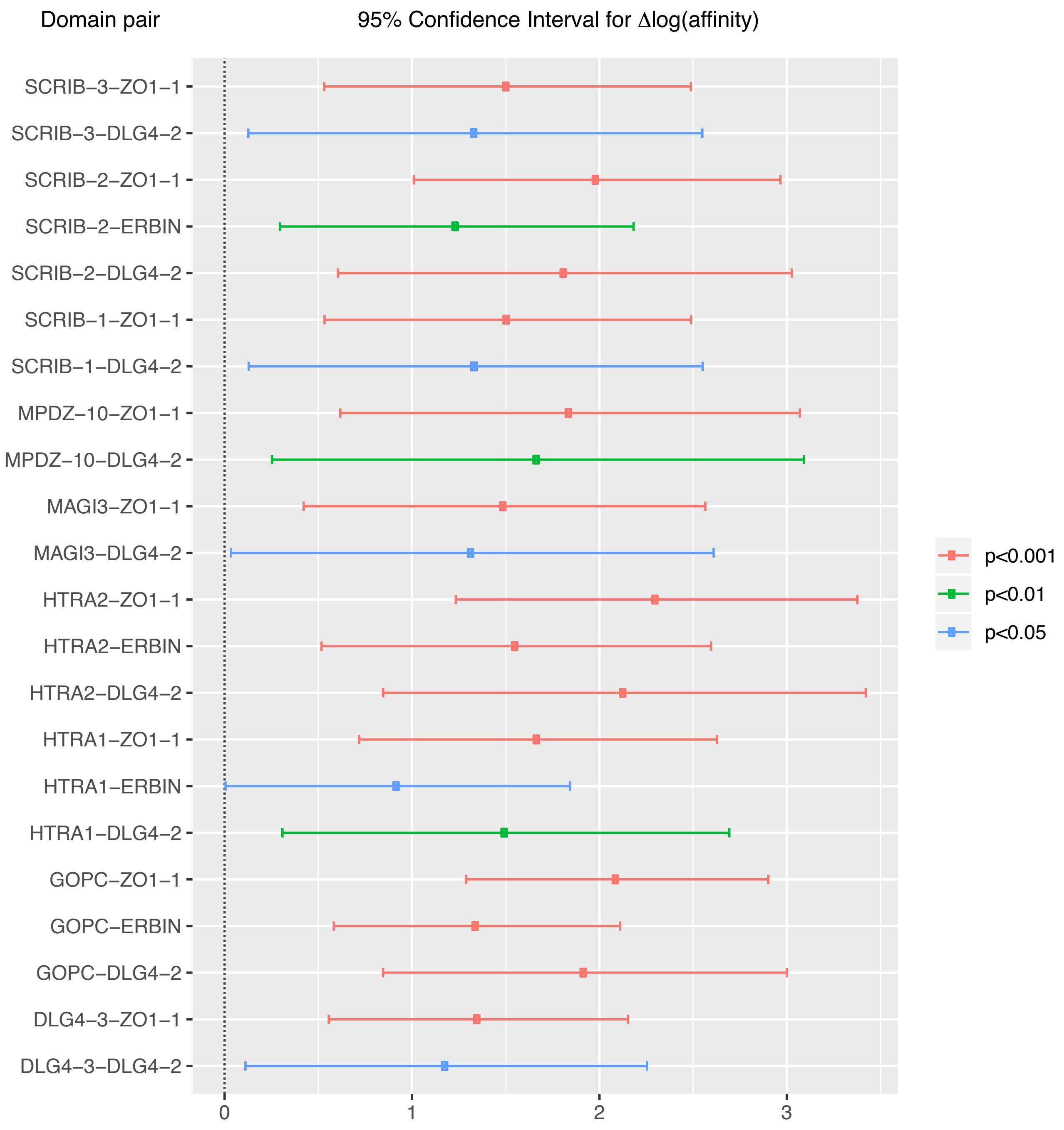

A two-way ANOVA was performed comparing $\log_{10}$-transformed affinity values for each pair of PDZ domains listed in Table S6. Tukey's HSD was computed to determine p_{adj} for each pair. Pairs with $p_{adj} < 0.05$ are shown, with the identity of each pair shown at left, and the 95% confidence interval for the difference in log(affinity) values shown as a bar colored by p_{adj} (blue, $p_{adj} < 0.05$; green, $p_{adj} < 0.01$; orange, $p_{adj} < 0.001$).
